# Rare variants in fox-1 homolog A (*RBFOX1*) are associated with lower blood pressure

**DOI:** 10.1371/journal.pgen.1006678

**Published:** 2017-03-27

**Authors:** Karen Y. He, Heming Wang, Brian E. Cade, Priyanka Nandakumar, Ayush Giri, Erin B. Ware, Jeffrey Haessler, Jingjing Liang, Jennifer A. Smith, Nora Franceschini, Thu H. Le, Charles Kooperberg, Todd L. Edwards, Sharon L. R. Kardia, Xihong Lin, Aravinda Chakravarti, Susan Redline, Xiaofeng Zhu

**Affiliations:** 1 Department of Epidemiology and Biostatistics, Case Western Reserve University, Cleveland, Ohio, United States of America; 2 Division of Sleep and Circadian Disorders, Brigham and Women’s Hospital, Boston, Massachusetts, United States of America; 3 Division of Sleep Medicine, Harvard Medical School, Boston, Massachusetts, United States of America; 4 McKusick-Nathans Institute of Genetic Medicine, Johns Hopkins University School of Medicine, Baltimore, Maryland, United States of America; 5 Division of Epidemiology, Department of Medicine, Institute for Medicine and Public Health, Vanderbilt Genetics Institute, Vanderbilt University, Nashville, Tennessee, United States of America; 6 Biosocial Methods Collaborative, Institute for Social Research, University of Michigan, Ann Arbor, Michigan, United States of America; 7 Department of Epidemiology, School of Public Health, University of Michigan, Ann Arbor, Michigan, United States of America; 8 Division of Public Health Sciences, Fred Hutchinson Cancer Research Center, Seattle, Washington, United States of America; 9 Department of Epidemiology, UNC Gillings School of Global Public Health, Chapel Hill, North Carolina, United States of America; 10 Department of Medicine, Division of Nephrology, University of Virginia, Charlottesville, Virginia, United States of America; 11 Department of Biostatistics, Harvard T.H. Chan School of Public Health, Boston, Massachusetts, United States of America; 12 Division of Pulmonary, Critical Care, and Sleep Medicine, Beth Israel Deaconess Medical Center, Boston, Massachusetts, United States of America; University of Pittsburgh, UNITED STATES

## Abstract

Many large genome-wide association studies (GWAS) have identified common blood pressure (BP) variants. However, most of the identified BP variants do not overlap with the linkage evidence observed from family studies. We thus hypothesize that multiple rare variants contribute to the observed linkage evidence. We performed linkage analysis using 517 individuals in 130 European families from the Cleveland Family Study (CFS) who have been genotyped on the Illumina OmniExpress Exome array. The largest linkage peak was observed on chromosome 16p13 (MLOD = 2.81) for systolic blood pressure (SBP). Follow-up conditional linkage and association analyses in the linkage region identified multiple rare, coding variants in *RBFOX1* associated with reduced SBP. In a 17-member CFS family, carriers of the missense variant rs149974858 are normotensive despite being obese (average BMI = 60 kg/m^2^). Gene-based association test of rare variants using SKAT-O showed significant association with SBP (p-value = 0.00403) and DBP (p-value = 0.0258) in the CFS participants and the association was replicated in large independent replication studies (N = 57,234, p-value = 0.013 for SBP, 0.0023 for PP). *RBFOX1* is expressed in brain tissues, the atrial appendage and left ventricle in the heart, and in skeletal muscle tissues, organs/tissues which are potentially related to blood pressure. Our study showed that associations of rare variants could be efficiently detected using family information.

## Author summary

Hypertension is a risk factor for cardiovascular disease and the most important risk factor for stroke. Family studies suggest that hypertension related traits are heritable. Previous genome-wide association studies (GWAS) have identified multiple common blood pressure (BP) variants but these variants do not overlap with the linkage evidence observed from family studies. Rare variants have been suggested to play a substantial role and contribute to missing heritability of BP. In this study, linkage analysis identified 16p13 linked to SBP in a cohort of 517 white individuals in 130 families from the Cleveland Family Study (CFS). By combining linkage and association analyses, we searched for rare, coding variants that can explain the linkage evidence. Rare, coding variants within *RBFOX1* were associated with lower systolic (p-value = 0.00403) and diastolic (p-value = 0.0258) blood pressures, and explained significant amount of observed linkage evidence. We replicated the identified variants in four independent cohorts (with total sample size N = 57, 234) and further observed consistent evidence that rare *RBFOX1* variants are protectively associated with blood pressure traits. Our study clearly shows that family-based designs are powerful for identifying rare, coding variants underlying complex diseases.

## Introduction

High blood pressure (BP) is a common condition associated with multiple health outcomes, including heart, brain, and kidney diseases [1, 2]. Previous studies have shown that BP is a genetically determined trait with estimated heritability of 30% to 60% [3, 4]. Multiple large genome-wide association studies (GWAS) meta-analysis and admixture mapping studies have identified over 190 genetic variants that explained only a small variation in BP [5–21].

For complex traits such as BP, rare variants are suggested to play a greater role in heritability than anticipated in the common disease-common variant hypothesis [22]. A Framingham Heart Study reported rare mutations in three renal salt handling genes causing large reductions in blood pressure and estimated that the overall prevalence of hypertension is reduced by about 1% because of the effects [23]. Linkage studies of family data can be used to uncover missing heritability and identify genetic markers linked to BP [24, 25]. However, the identified linkage regions from well-designed linkage studies such as the US Family Blood Pressure Program (FBPP) and the UK Medical Research Council British Genetics of Hypertension (BRIGHT) study [26–29] do not overlap with many BP loci identified by large BP GWAS of mostly unrelated individuals. In general, GWAS have good power to detect common variants of modest effect with attainable sample sizes, but less power for detecting rare variants with intermediate effect. In contrast, linkage analysis can have good power to detect multiple rare or lower frequency BP variants in a gene or region with relatively larger effect sizes [25]. Thus, we hypothesize that a linkage region observed in a family study, if not overlapping with the BP loci in reported GWAS, may harbor multiple rare or lower frequency BP variants.

Recently, many statistical approaches for rare variant association analyses have been developed for unrelated samples [30–34] and family data [35–38]. It has been suggested that rare or lower frequency variants can be enriched in families [35, 37], and therefore improving the statistical power for their identification. However, the existing rare variant association methods have not incorporated linkage evidence. In this study, we performed variance-component linkage analysis with BP traits, including systolic blood pressure (SBP), diastolic blood pressure (DBP), and pulse pressure (PP) in the Cleveland Family Study (CFS). We searched the published GWAS to examine whether there are reported BP variants in the linkage regions. Using the combined linkage and association analyses, we searched for potential functional variants that can explain linkage evidence and replicated the variants in independent cohorts.

## Results

**Fig 1:**
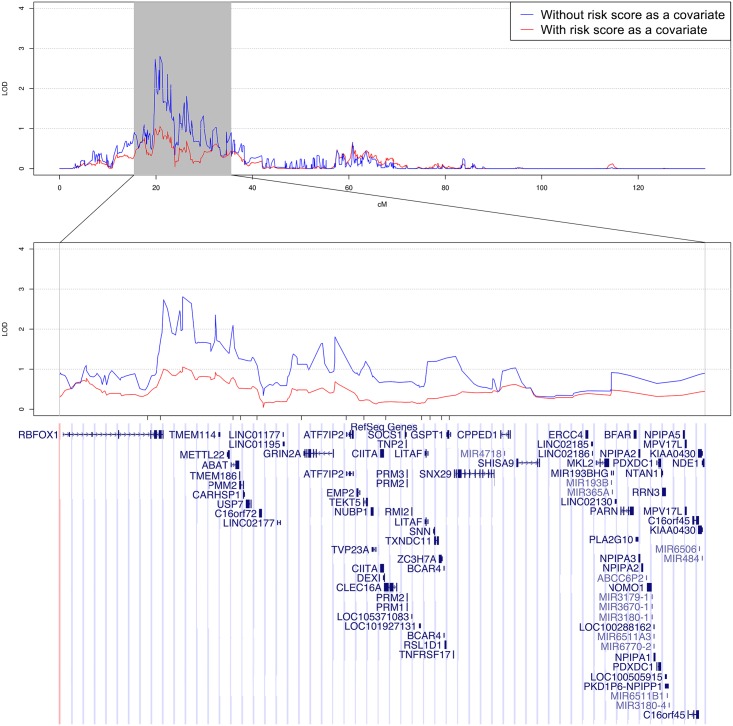
Linkage region on chromosome 16 of white participants in CFS. Linkage peak on chromosome 16 for SBP. The linkage curves are plotted with (red curve) and without (blue curve) adjusting for the risk score defined by the 13 coding variants as a covariate. The positions of the 13 coding variants are listed under the linkage peak and above the genes.

Table 1 presents the characteristics of white participants in the CFS data. S1 Fig presents the distributions of the residuals of SBP, DBP, and PP, which are all approximately normally distributed. Linkage analysis identified a peak (LOD = 2.81) on chromosome 16p13 linked to SBP (S2 Fig, Materials and Methods). Linkage analysis of further pruned markers using a R^2^ threshold of 0.1 or modeling marker-marker linkage disequilibrium resulted in a slight decrease of LOD score in the same region (LOD = 2.30–2.42, S3 Fig). We selected a candidate region of 20cM with 2-LOD score drop for association analysis (Fig 1). This region did not overlap with published GWAS of BP variants. Therefore, we tested the hypothesis that the observed linkage evidence for SBP is due to the presence of multiple rare, coding variants in a gene(s) within the region.

**Table 1:** Characteristics of white participants in the Cleveland Family Study.

	Mean ± SD or N (%)
Individuals	517
Families	130
Male	236 (45.6%)
Age (years)	46.9 ± 16.6
BMI (kg/m^2^)	31.8 ± 8.55
SBP (mm Hg)	123.7 ± 15.2
SBP (medication adjusted)	125.3 ± 16.6
DBP (mm Hg)	73.4 ± 9.70
DBP (medication adjusted)	74.5 ± 10.6
PP (mm Hg)	50.3 ± 12.2
PP (medication adjusted)	50.8 ± 12.5

The CFS was genotyped by an exome array, with most of the variants being coding variants. Within the linkage region, there are 454 exonic variants defined by the Cohorts for Heart and Aging Research in Genomic Epidemiology (CHARGE) consortium that are genotyped on the exome array [39]. We identified 13 exonic variants (S1 Table) that satisfy the following filtering criteria: 1) either have a SBP association p-value ≤ 0.1 or absolute regression coefficient beta ≥ 5; and 2) present at least twice in at least one family with a family-specific LOD score ≥ 0.1. A risk score based on the 13 identified SNPs has an effect size of 0.948 ± 0.135 for association with SBP in CFS. After adding this risk score as a covariate in linkage analysis, the MLOD score dropped from 2.809 to 1.055, suggesting that these 13 variants were able to account for most of the observed linkage evidence. To further assess the significance of this LOD drop, we sampled 1,000 independent SNPs from chromosomes other than chromosome 16. Hence, these SNPs should not contribute to the LOD score observed on chromosome 16. We performed linkage analysis with each of these 1,000 SNPs as a covariate in the linkage analysis. We calculated the differences between the original MLOD score and the MLOD scores of the 1,000 linkage analyses with a SNP as a covariate. The largest LOD score drop in these 1,000 linkage analyses was 0.347, suggesting the observed LOD score drop on the risk scores of 13 selected variants is statistically significant (p-value<0.001). Among these 13 exonic variants, two variants (rs149974858 and rs145873257) are present in *RBFOX1* and the remaining 11 variants are each in separate genes. When adjusting for the risk scores of rs149974858 and rs145873257, we also observed a drop in LOD score (LOD = 1.97), which suggests that rs149974858 and rs145873257 account for a portion of the observed linkage evidence.

**Fig 2:**
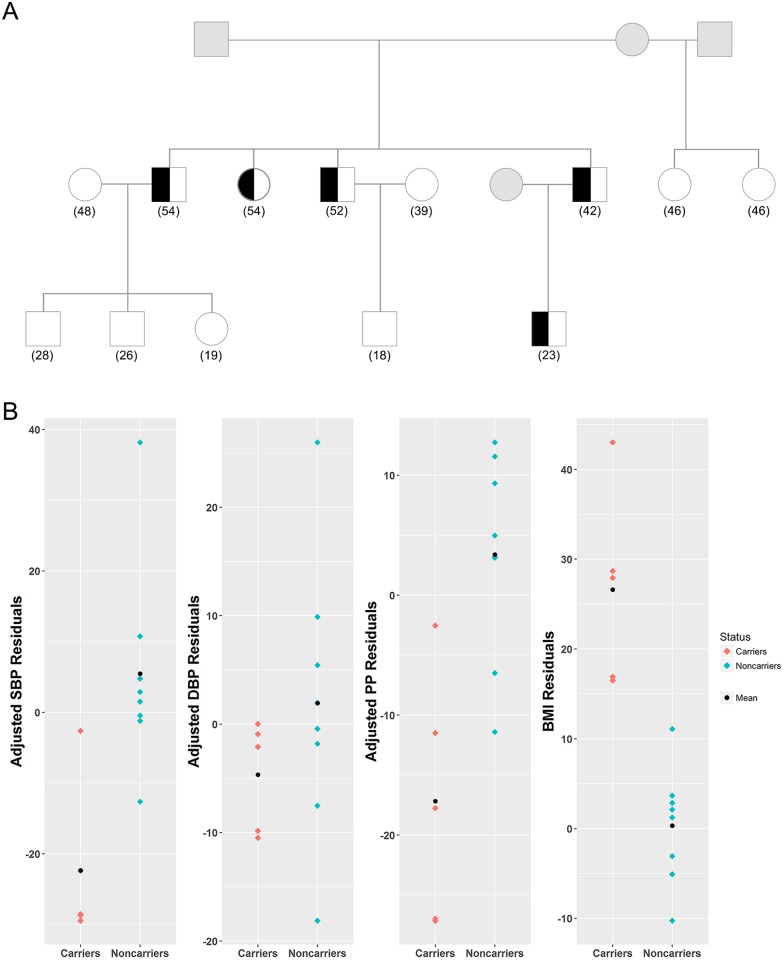
The CFS family carrying the protective rare variant rs149974858. A) The variant rs149974858 segregates with BP in a 17-member CFS family. Squares represent males and circles represent females. Half-filled subjects represent the carriers of the rare variant rs149974858. Grey subjects represent no information. Age of each subject is presented in parenthesis. B) The distribution of corresponding residuals of SBP, DBP, PP and BMI are presented.

The variant rs149974858 shows association evidence with SBP (p-value = 0.0016) in CFS. The minor allele frequency of rs149974858 is 0.0036 and only segregates within a 17-member family with family-specific LOD of 0.697 (Fig 2). This missense variant (c.112C>G) results in a proline to alanine substitution (p.Pro38Ala). Five members from this family carrying this rare missense mutation had on average lower SBP (carrier average = 117 mmHg, noncarrier average = 125 mmHg) but higher BMI than other family members (carrier average = 60 kg/m^2^, noncarrier average = 31 kg/m^2^). A single SNP association test revealed that rs149974858 is also significantly associated with BMI in CFS (beta = -26.8±4.35, asymptotic p-value = 7.28E-10). The exonic variant rs145873257 segregates within a different family with family-specific LOD score of 0.215. A c.1072G>A base change resulted in a glycine to serine substitution (p.Gly358Ser). Six members of this family are heterozygous for the AT genotype. The estimated effect of this variant is protective in CFS, although it is not statistically significant (S1 Table).

Since both variants rs149974858 and rs148751394 consistently show a protective effect in two large families, we examined the other coding and rare variants in *RBFOX1* genotyped on the exome array. Two exonic variants, rs151214012 and rs145873257, and one rare, intronic variant rs2345080 are available in the exome array. These three variants show protective effect despite not satisfying the filtering criteria (S2 Table). Single SNP associations and annotations for all exome array variants of *RBFOX1* are provided in the S3 Table. Applying either the family-based burden or SKAT analysis, these five variants are significantly associated with SBP and DBP (Table 2).

**Table 2:** Results of gene-based analysis in discovery data and replication cohorts.

	Methods	CFS	ARIC	WHI	BioVU	HRS	Meta of replication cohorts ^a^
**SBP**							
Rare variants	Burden	**2.14E-3**	5.72 E-3	6.68E-1	3.44E-1	6.95E-2	**1.71E-2**
	SKAT	7.02E-2	2.59E-3	6.71E-1	1.13E-1	1.19E-1	**6.35E-3**
	SKAT-O	**4.03E-3**	3.56E-3	8.14E-1	1.79E-1	1.15E-1	**1.26E-2**
**DBP**							
Rare variants	Burden	**1.48E-2**	3.28E-1	4.08E-1	9.44E-1	1.20E-1	3.98E-1
	SKAT	**4.94E-2**	4.61E-1	7.58E-1	3.98E-2	3.01E-1	2.05E-1
	SKAT-O	**2.58E-2**	4.75E-1	5.71E-1	6.42E-2	2.41E-1	2.05E-1
**PP**							
Rare variants	Burden	8.13E-2	1.40E-3	2.31E-1	2.52E-1	1.44E-1	**3.77E-3**
	SKAT	1.27E-1	2.73E-4	5.11E-1	4.23E-1	1.27E-1	**2.66E-3**
	SKAT-O	1.28E-1	3.92E-4	3.45E-1	3.80E-1	1.23E-1	**2.34E-3**
**BMI**							
Rare variants	Burden	4.24E-3	4.35E-1	N/A	6.40E-1	7.26E-1	7.84E-1
	SKAT	4.28E-4	6.50E-1	N/A	7.80E-1	7.26E-1	9.20E-1
	SKAT-O	7.32E-4	6.04E-1	N/A	8.07E-1	5.07E-1	8.34E-1
Number of rare variants		5	8	11	8	11	

^a^ Meta-analysis of ARIC, WHI, BioVU, and HRS

We next sought the replication of the rare variants in *RBFOX1* in four large independent cohorts (ARIC, WHI, BioUV, and HRS) of whites for the traits SBP, DBP and PP. We specifically looked at the five rare variants of *RBFOX1* found in CFS and their associations with BP traits. Within the ARIC data, 4 of the 5 variants were present and all the 4 variants showed a consistent protective effect. For WHI, all 5 variants were present but only 1 variant (rs145873257) had a protective effect size. BioUV contained 4 out of 5 variants found in CFS; 2 variants were protective for SBP and 3 variants were protective for DBP. HRS contained all 5 variants found in CFS; 4 variants were protective for SBP and 3 variants were protective for DBP. Among the total 23 tests (CFS: 5, ARIC: 4, BioUV: 4, WHI: 5, HRS: 5) conducted across all cohorts, 16 of them were protective (p-value = 0.0173 based on binomial distribution), suggesting a consistent protective effect.

We next conducted a gene-based association analyses for *RBFOX1* and BP traits using exome array data from the CFS with weights Beta (1, 25). Burden, SKAT, and SKAT-O tests were performed using the 5 rare variants of *RBFOX1* found in CFS (Table 2). In CFS, the association between *RBFOX1* and SBP was found to be significant by the burden test (p-value = 0.00214) and SKAT-O (p-value = 0.00403), but not by SKAT (p-value = 0.0702). All three gene-based tests for DBP were statistically significant (burden test p-value = 0.0148, SKAT p-value = 0.0494, SKAT-O p-value = 0.0258). When we conducted gene-based analyses using only the 4 coding variants (rs149974858, rs148751394, rs151214012, rs145873257) identified in CFS, the associations are also significant for SBP and DBP (p-value<0.037).

The same gene-based analysis for rare variants was done for all four replication cohorts separately and the results were meta-analyzed (Table 2). Individually, the ARIC cohort had significant gene-based associations for SBP (burden test p-value = 0.00572, SKAT p-value = 0.00259, SKAT-O p-value = 0.00356) and PP (burden test p-value = 0.00140, SKAT p-value = 0.000273, SKAT-O p-value = 0.000392). After meta-analyzing the results for ARIC, WHI, BioVU, and HRS, we found significant associations for SBP (burden test p-value = 0.0172, SKAT p-value = 0.00635, SKAT-O p-value = 0.0126) and PP (burden test p-value = 0.00377, SKAT p-value = 0.00266, SKAT-O p-value = 0.00234).

We observed that the variant rs149974858 co-segregated with BMI in the 17-member CFS family. Subsequently, we performed linkage analysis for BMI in CFS on chromosome 16, after adjusting for gender, age, age^2^, and PC1. We did not observe linkage evidence in this region (LOD = 0.721). The gene-based analysis of BMI using the same set of variants was only significant in CFS but not in any of the replication cohorts (Table 2).

## Discussion

We performed a linkage analysis of BP traits using the families collected in CFS. The largest linkage peak identified is on 16p13 linked to SBP. The 16p13 region has been reported of linkage evidence with BP in two studies: the Victorian Family Heart Study [40], the extreme-sib-pair study in Chinese adults [41]. In addition, the longitudinal change of BP in Mexican Americans [42], and the Hypertension Genetic Epidemiology Network Study in whites [43] reported linkage regions partially overlapped with the current study. The reported linkage evidence from multiple ethnic populations strongly suggests the linkage evidence on 16p13 is real. Linkage analysis using microsatellite markers was performed with 363 sib-pairs of CFS whites for a hypertensive status, adjusted for age, age^2^, sex, BMI, and BMI^2^. This analysis did not find linkage evidence in the 16p13 region. This is unsurprising because the power of using a binary hypertensive status is lower than that of quantitative phenotypes, such as SBP, DBP, or PP. In addition, hypertensive status was defined as either SBP ≥ 140, DBP ≥ 90, or taking antihypertensive medications and the sample size in the sib-pair analysis was smaller than the current study, all of which contribute to the lack of linkage evidence observed. Our study demonstrates that high-density SNP genotyping arrays are informative for detecting linkage signals.

We searched among published large GWAS studies of BP traits [5, 7–10] and did not identify any BP variants previously reported on 16p13, suggesting that multiple low frequency or rare variants with relatively large effect sizes are possibly contributing to the observed linkage evidence. We further assumed that variants with relatively large effect sizes are more likely to be coding and rare variants. Thus, we limited our search to only the coding and rare variants under the linkage peak genotyped on the exome array. By examining the associated variants that are able to account for the observed linkage evidence on 16p13, we were able to identify 13 exonic variants. Among these 13 variants, 2 of them fall in *RBFOX1*, which encodes for the ataxin-2 binding protein 1 (also known as *A2BP1*), and show a consistent protective effect for SBP in CFS. Gene-based analysis of the four available exonic variants and one rare intronic variant in *RBFOX1* are significantly associated with SBP and DBP (p-value = 0.00403, 0.0258, respectively) using SKAT-O. Replication analysis of the rare variants at the gene level (but not at the variant level) is also significant for SBP and PP in the meta-analysis of four large cohorts of whites with a total replication sample size N = 57,234.

This study also provides evidence that rare variants within *RBFOX1* are protective for BP traits among obese individuals. Among individuals of European ancestry within the CFS, 5 individuals within the same family carried the minor allele for rs149974858, the variant showing significantly protective effect with SBP by single SNP association test. All of the 5 individuals are morbidly obese (with average BMI of 60). However, their SBP (mean = 117) and DBP (mean = 78) are within the normotensive range. Ma et al. conducted a GWAS of BMI in Pima Indians using Affymetrix 100K array and identified two common variants in *RBFOX1* associated with BMI [44]. The same two variants could be replicated in non-overlapped Pima Indians but not in French Caucasians, Amish Caucasians, German Caucasians, or Native Americans [44]. In our analysis, we identified four exonic and one intronic rare variants in *RBFOX1* that are significantly associated with BMI but the association evidence could not be replicated (Table 2). Therefore, it is inconclusive whether *RBFOX1* is an obesity gene. In all our analysis, either linkage or association analysis, BMI is included as a covariate. Furthermore, no linkage evidence was found for BMI on chromosome 16, after adjusting for gender, age, age^2^, and PC1. Our result indicates the *RBFOX1* contributes to BP variation independent of obesity, although we are unclear whether *RBFOX1* has a pleiotropic effect on both BP and obesity.

We also observed that the effect direction of single variant replication analysis in the four cohorts is not always consistent with that of CFS. However, 16 of the 23 tests were protective (p-value = 0.017), suggesting a consistent protective effect. Assuming a causal rare variant with an effect size equal to one quarter of the BP standard deviation, we estimate the probability of observing an opposite direction in a study to be 40.1%, which is consistent with 7 opposite directions among 18 single SNP replication tests in 4 replication cohorts.

It is interesting that all the four exonic variants in *RBFOX1* are either monomorphic or extremely rare in African ancestry populations (S4 Table). Furthermore, the BP admixture mapping analysis by Zhu et al. also suggest local ancestry in this region is associated with SBP and DBP; however, the evidence is not genome-wide significant [11]. Thus, our result suggests that the rare exonic variants in *RBFOX1* may contribute to a protective effect for hypertension and further work will be needed to establish whether the lack of these protective variants contribute to the disparity in hypertension occurrence and early age of onset between African Americans and whites.

To our knowledge, only one GWAS study so far has reported on the association of *RBFOX1* variants with blood pressure using human genotyping data. Wang et al. reported an association between rs1507023, a candidate SNP in *RBFOX1* involved in vitamin D metabolism and signaling, and SBP, DBP, and PP. Its association with blood pressure was significant before, but not after correction for multiple testing [45].

Under the linkage region of 16p13, there are 11 additional variants that either have an association test p-value less than 0.1 or an effect size larger than 5 mmHg in CFS. When we used the risk scores of these 11 variants as a covariate in linkage analysis, the MLOD dropped to 1.932, suggesting that there may be additional variants that contribute to linkage evidence in this region. However, the current exome array data is limited for further dissection of genes or variants contributing the linkage evidence. Whole genome sequencing data, including the sequencing data from the Trans-Omics for Precision Medicine (TOPMed) Program (https://www.nhlbi.nih.gov/research/resources/nhlbi-precision-medicine-initiative/topmed), will be necessary to identify the genes contributing blood pressure variation in this region.

**Fig 3:**
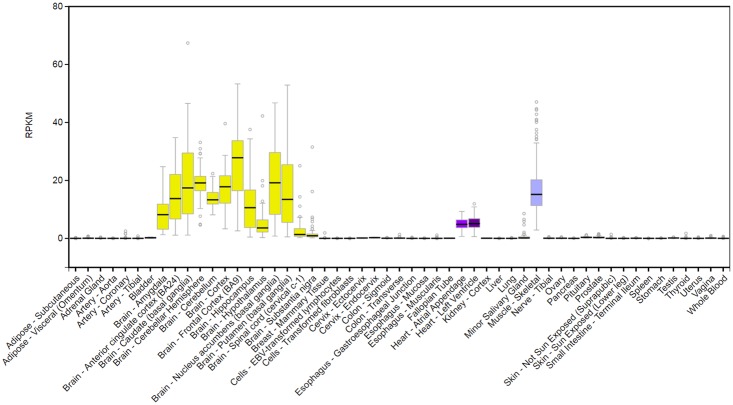
Tissue-specific gene expression of *RBFOX1* from GTEx database (http://www.gtexportal.org/home/gene/RBFOX1).

Gene expression data and previous studies have demonstrated that RNA splicing factor *RBFOX1* is important for heart and skeletal muscle development and function [46–48]. *RBFOX1* expression has been associated with cardiac hypertrophy and heart failure in mice models [49]. Gao et al. found that *RBFOX1* expression was significantly diminished in both mouse and human failing hearts [49]. We searched the GTEx database and *RBFOX1* is highly expressed in multiple human brain tissues, atrial appendage and left ventricle of the heart, as well as muscle skeletal tissues (Fig 3; http://www.gtexportal.org/home/gene/RBFOX1). Further biological studies are needed to establish the direct role of *RBFOX1* in regulating blood pressure.

Our study suggests that family-based linkage evidence can be extremely successful in searching for rare variants contributing to complex traits. In summary, we identified rare, exonic variants in *RBFOX1* that have a protective effect on BP traits, which can be important in searching new drugs for cardiovascular disease. However, it should be pointed out that association analysis was performed using variants available in the exome array of this study. The variants identified in *RBFOX1* may still be reflecting in LD with the causal variants to BP. While *RBFOX1* is expressed in multiple tissues that may relate to blood pressure, the mechanism underlying how this gene contributes to BP variation needs to be further studied. The identification of these rare coding variants will facilitate precision medicine in treating cardiovascular disease.

## Materials and methods

The CFS is a family-based longitudinal study comprised of patients with laboratory-diagnosed sleep apnea, their family members, and neighborhood control families, as described before [50]. The data were analyzed anonymously at Case Western Reserve University. The CFS study was approved by Partners Human Research Committee with the proposal number 2011D001860. The analytical sample includes 517 white participants in 130 families who were genotyped with the Illumina OmniExpress Exome array, which includes both GWAS and exome variants (Table 1). Standard quality controls were performed, including checking Hardy-Weinberg equilibrium, Mendelian inconsistences, and verifying pedigree structure using the genetic markers by the software PLINK [51]. BP traits, including SBP and DBP were each determined following standardized guidelines using a calibrated sphygmomanometer [52]. Height and weight were directly measured and antihypertensive medications were ascertained by questionnaire. Data for this analysis were from the last available examination for each participant.

The samples used for replication analysis include five independent cohorts. We included 10,864 unrelated white participants from the Atherosclerosis Risk in Communities (ARIC) Study. The ARIC study is a prospective epidemiologic study designed to investigate the natural history and etiology of atherosclerosis (https://www2.cscc.unc.edu/aric/). There were 18,050 unrelated white participants from the Women’s Health Initiative (WHI), a study of postmenopausal women focused on strategies for preventing heart disease, breast and colorectal cancer, and osteoporotic fractures [53, 54]. From the Vanderbilt University Biobank (BioVU), we included 18,977 unrelated white individuals. BioVU uses leftover blood samples collected during routine clinical care from consented individuals who visit the Vanderbilt University Medical Center [55]. Lastly, we included 9,343 unrelated white participants from the Health and Retirement Study (HRS). This is a longitudinal survey of a representative sample of Americans over the age of 50 [56–58].

SBP and DBP for an individual taking antihypertensive medication were imputed using the standard approach in literature, by adding 15 mmHg and 10 mmHg, respectively. Pulse pressure (PP) was calculated as the difference between imputed SBP and DBP [5].

### Linkage analysis in CFS

We calculated residuals of SBP, DBP, and PP after adjusting for gender, age, age^2^, body mass index (BMI; kg/m^2^) and the first principal component of genotype values in CFS separately. The residuals of these regressions were used for linkage analysis using the software MERLIN [59]. The principal components (PCs) were calculated using the software FamCC, which can be applied to family data [60]. Since the results were essentially the same for including the first PC or the first 10 PCs, we reported the linkage results including the first PC. We used the pairwise linkage disequilibrium (LD) pruning approach with a window size of 50 kb, step size of 5 variants, and R^2^ threshold of 0.2. We also required a minor allele frequency (MAF) ≥ 0.2. This resulted in 56,992 autosomal SNPs using PLINK [51]. Because marker-marker LD may result in biased linkage calculations, we performed linkage analysis by further reducing the R^2^ threshold to 0.1 and by modeling the marker-marker LD using MERLIN [59]. Linkage analysis using MERLIN decomposes phenotypic variance into three parts: the variance contributes to the quantitative trait locus ($\sigma_{QTL}^{2}$), the variance contributes to the polygenetic effect ($\sigma_{G}^{2}$), and the variance contributes to the random effect ($\sigma_{E}^{2}$). It also tests the null hypothesis of no linkage $H_{0}:\sigma_{QTL}^{2}=0$ vs. $H_{A}:\sigma_{QTL}^{2}>0$.

### Identify coding variants accounting for linkage evidence

We examined exonic variants genotyped in the exome array in the region of 2-LOD score drop from the linkage peak. We performed the family-based association analysis for the exonic variants only in the 2-LOD drop region using the ASSOC package in S.A.G.E [61]. The family-based association analysis was conducted using a linear mixed model *y* = *β*_0_ + *β*_1_*g + δ* + *ε*, where *g* is a genotype value vector, *β*_0_ is the intercept, *β*_1_ is the regression coefficient, $\delta \;\sim \;N\left(0,\;2\Phi \sigma_{G}^{2}\right)$ where *Φ* is the kinship coefficient matrix and $\sigma_{G}^{2}$ is the polygenic variance, and *$\varepsilon \;\sim \;N\left(0,\;I\sigma_{\varepsilon }^{2}\right)$* where $\sigma_{\varepsilon }^{2}$ is the random error. ASSOC applies the likelihood ratio test to test the null hypothesis of *H*_0_: *β*_1_ = 0. For each of the variants, we first performed an association analysis with a BP trait using ASSOC and identified variants with either p-value ≤ 0.1 (marginal effect) or absolute regression coefficient beta ≥ 5 (large effect). We next estimated family-specific LOD scores and identified families with LOD score ≥ 0.1. We kept the variants with association p-value ≤ 0.1 or absolute regression coefficient ≥ 5, and that were present at least twice in at least one family with family-specific LOD score ≥ 0.1.

We defined the risk score as $r_{i}=x_{i}^{T}\beta$, where *β* is the regression coefficients of the SNPs, and *x*_*i*_ is a vector of the number of risk alleles carried by individual *i* for these SNPs. Linkage analysis was further performed conditional on the risk scores.

### SNP-set burden and SKAT test

We performed family-based burden and SKAT tests for CFS using the software famSKAT and for the replication cohorts using the R package SKAT [30, 32, 62]. The weight was set to $\sqrt{w_{j}}=Beta\left(MAF_{j},1,25\right)$ as suggested to increase the weight of rare variants.

## Supporting information

S1 Fig The distributions of residuals of SBP, DBP, and PP in CFS. (TIFF)

S2 Fig Linkage results of SBP, DBP, and PP in CFS. (TIF)

S3 Fig Linkage analysis by additional LD-based pruning or modeling marker-marker LD for chromosome 16. Original (blue): pairwise LD pruning with a window size of 50 kb, step size of 5 variants, and R^2^ threshold of 0.2; MAF ≥ 0.2. Further pruning (purple): pairwise LD pruning with a window size of 50 kb, step size of 5 variants, and R^2^ threshold of 0.1; MAF ≥ 0.3. Cluster (red): modeling marker-marker LD using “—cluster” option with R^2^ threshold of 0.1; all other parameters are the same as the original linkage analysis.
(TIFF)

S1 Table Single association analysis of 13 exonic variants within the linkage region. ^a^ Include both founders and nonfounders
(DOCX)

S2 Table Single SNP association effect sizes and p-values for SBP, DBP, PP. (DOCX)

S3 Table Single SNP association analysis for exome array variants of *RBFOX1* identified in CFS. ^a^ Include both founders and nonfounders
(DOCX)

S4 Table Minor allele frequencies ^a^ of *RBFOX1* rare coding variants in white, African-American, and African populations. ^a^ Include both founders and nonfounders.
^b^ Calculated by the weighted average of Cleveland Family Study, Atherosclerosis Risk in Communities, Women’s Health Initiative, Vanderbilt University Biobank, and Health and Retirement Study.
^c^ Calculated by the weighted average of Atherosclerosis Risk in Communities, Family Blood Pressure Program, Africa America Diabetes Mellitus Study, and Howard University Family Study (H.W., unpublished data).
^d^ Calculated based on exome array of Nigeria data (H.W., unpublished data).
(DOCX)
