# Supplementary figures and images for: Rare variants in fox-1 homolog A (RBFOX1) are associated with lower blood pressure
Source: PLoS Genet. 2017 Mar 27;13(3):e1006678. doi: 10.1371/journal.pgen.1006678 (PMC5386302; doi:10.1371/journal.pgen.1006678)

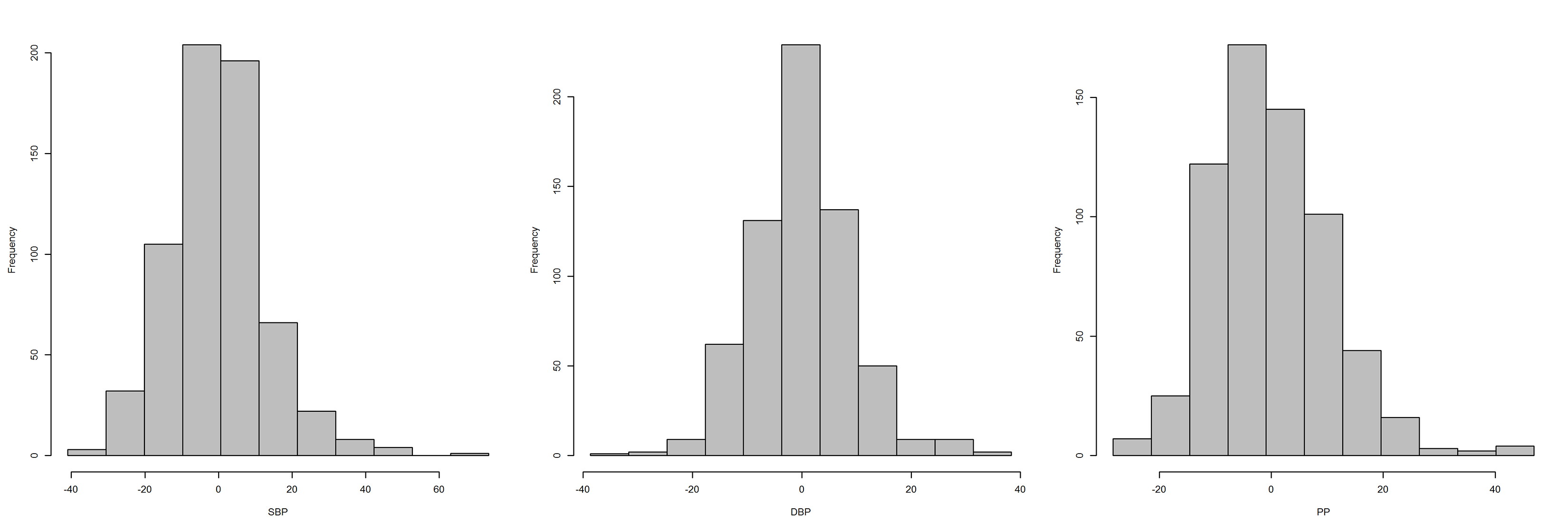

Supplement: S1 Fig — (TIFF) [file pgen.1006678.s001.tiff]

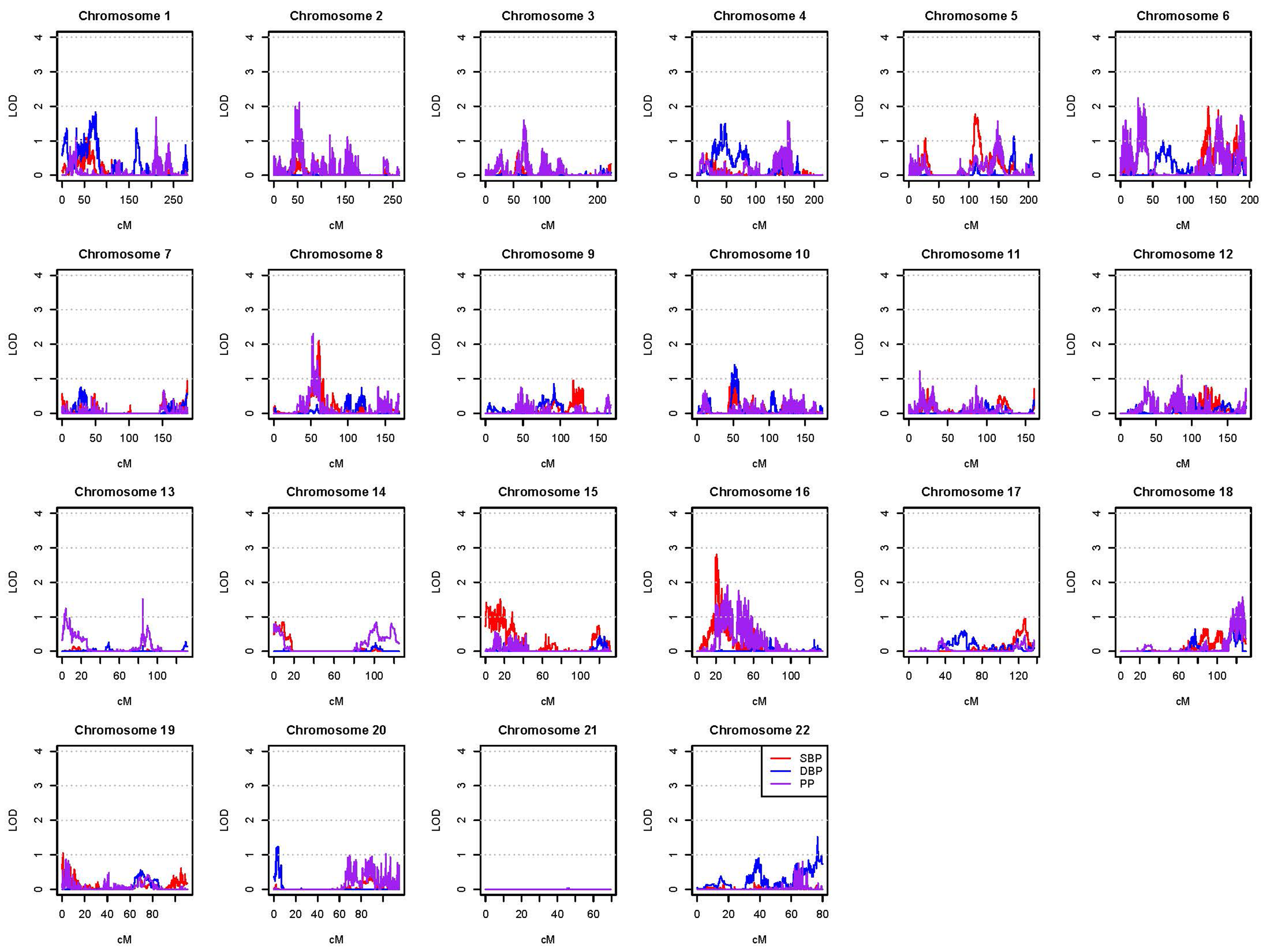

Supplement: S2 Fig — (TIF) [file pgen.1006678.s002.tif]

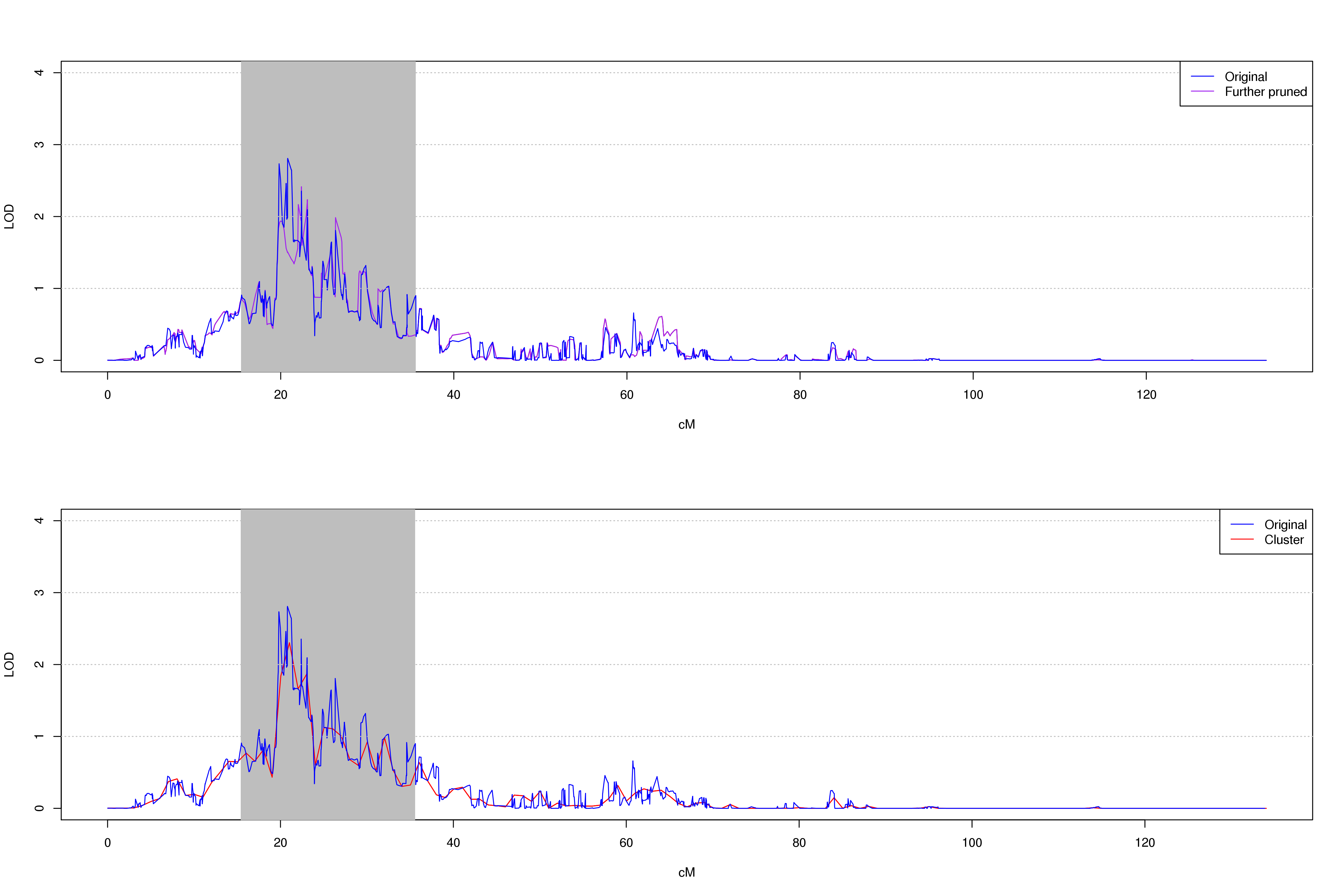

Supplement: S3 Fig — Original (blue): pairwise LD pruning with a window size of 50 kb, step size of 5 variants, and R2 threshold of 0.2; MAF ≥ 0.2. Further pruning (purple): pairwise LD pruning with a window size of 50 kb, step size of 5 variants, and R2 threshold of 0.1; MAF ≥ 0.3. Cluster (red): modeling marker-marker LD using “—cluster” option with R2 threshold of 0.1; all other parameters are the same as the original linkage analysis. (TIFF) [file pgen.1006678.s003.tiff]
