# Supplementary material for: Rare variants in fox-1 homolog A (RBFOX1) are associated with lower blood pressure
Source: PLoS Genet. 2017 Mar 27;13(3):e1006678. doi: 10.1371/journal.pgen.1006678 (PMC5386302; doi:10.1371/journal.pgen.1006678)
Supplement: S1 Table — a Include both founders and nonfounders (DOCX) [file pgen.1006678.s004.docx]

**S1 Table. Single association analysis of 13 coding variants within the linkage region.**

| **SNP** | **Gene** | **MAF ^a^** | **MAF ^b^** | **Estimate** | **SE** | **P-Value** |
| --- | --- | --- | --- | --- | --- | --- |
| rs149974858_G | *RBFOX1* | 3.59E-3 | 0 | -2.12E+1 | 6.63 | 1.58E-3 |
| rs145873257_A | *RBFOX1* | 8.61E-3 | 1.10E-2 | -5.26 | 3.86 | 1.74E-1 |
| rs1731017_A | *ABAT* | 4.00E-1 | 4.12E-1 | 1.27 | 7.20E-1 | 7.75E-2 |
| rs149816371_T | *CARHSP1* | 2.89E-3 | 2.75E-3 | 1.34E+1 | 6.24 | 3.15E-2 |
| rs137974792_A | *C16orf72* | 5.02E-3 | 5.50E-3 | 1.12E+1 | 4.92 | 2.40E-2 |
| rs61731465_G | *GRIN2A* | 6.46E-3 | 8.24E-3 | 5.00 | 4.32 | 2.48E-1 |
| rs74322853_G | *ATF7IP2* | 5.02E-3 | 2.75E-3 | 7.04 | 5.58 | 2.06E-1 |
| rs145995713_C | *TEKT5* | 8.61E-3 | 5.50E-3 | 9.40 | 3.70 | 1.13E-2 |
| rs74163614_T | *CLEC16A* | 3.59E-3 | 2.75E-3 | 8.37 | 5.79 | 1.48E-1 |
| rs4280262_C | *LITAF* | 2.35E-1 | 2.42E-1 | 1.54 | 8.10E-1 | 5.91E-2 |
| rs143228508_A | *TXNDC11* | 1.44E-3 | 0 | -1.51E+1 | 8.71 | 8.31E-2 |
| rs150419519_A | *RSL1D1* | 3.59E-3 | 2.75E-3 | -8.18 | 5.95 | 1.70E-1 |
| rs373496_A | *TNFRSF17* | 1.08E-2 | 2.75E-3 | 5.14 | 3.27 | 1.16E-1 |

^a^ Include both founders and nonfounders

^b^ Founders only; MAF=0 indicates that founders for carriers are not genotyped
