# Supplementary material for: Rare variants in fox-1 homolog A (RBFOX1) are associated with lower blood pressure
Source: PLoS Genet. 2017 Mar 27;13(3):e1006678. doi: 10.1371/journal.pgen.1006678 (PMC5386302; doi:10.1371/journal.pgen.1006678)
Supplement: S2 Table — (DOCX) [file pgen.1006678.s005.docx]

**S2 Table. Single SNP association effect sizes and p-values for SBP, DBP, PP.**

|  |  | **SBP** | | **DBP** | | **PP** | |
| --- | --- | --- | --- | --- | --- | --- | --- |
| **SNP** | **Cohort** | **Estimate** | **P-Value** | **Estimate** | **P-Value** | **Estimate** | **P-Value** |
| rs2345080 (16:6602397) | CFS | -3.26 | 2.58E-1 | -3.62 | 7.31E-2 | 4.30E-1 | 8.55-1 |
|  | ARIC | -2.01 | 1.19E-2 | -5.29E-1 | 2.78E-1 | -1.49 | 6.10-3 |
|  | WHI | 5.37E-1 | 4.18E-1 | -4.72E-2 | 8.95E-1 | 5.84E-1 | 2.29-1 |
|  | BioVU | N/A | N/A | N/A | N/A | N/A | N/A |
|  | HRS | -1.35 | 1.80E-1 | -5.10E-1 | 3.80E-1 | -8.40E-1 | 1.90-1 |
| rs149974858  (16:7568173) | CFS | -2.22E1 | 1.55E-3 | -3.32 | 4.83E-1 | -1.81E1 | 1.60E-3 |
|  | ARIC | -1.46 | 7.57E-1 | 1.30 | 6.53E-1 | -2.75 | 3.92E-1 |
|  | WHI | 4.72 | 3.89E-1 | -5.57E-1 | 9.85E-1 | 4.77 | 2.34E-1 |
|  | BioVU | -3.67 | 4.00E-1 | 1.81E-1 | 9.44E-1 | -3.85 | 2.49E-1 |
|  | HRS | 9.87 | 7.00E-2 | 6.71 | 3.00E-2 | -3.16 | 3.50E-1 |
| rs148751394  (16:7568191) | CFS | -1.42E1 | 2.48E-1 | -3.92 | 6.60E-1 | -9.61 | 3.35E-1 |
|  | ARIC | N/A | N/A | N/A | N/A | N/A | N/A |
|  | WHI | 4.72 | 3.89E-1 | -5.57E-2 | 9.85E-1 | 4.77 | 2.34E-1 |
|  | BioVU | -1.02E1 | 6.00E-1 | -6.03 | 6.00E-1 | -4.21 | 7.78E-1 |
|  | HRS | -4.77E1 | 2.00E-2 | -1.05E1 | 3.90E-1 | -3.72E1 | 4.00E-3 |
| rs151214012  (16:7568263) | CFS | -1.98E1 | 1.20E-1 | 8.48 | 3.49E-1 | -2.59E1 | 1.25E-2 |
|  | ARIC | -1.19 | 7.12E-1 | -1.45E-1 | 9.42E-1 | -1.08 | 6.23E-1 |
|  | WHI | 4.72 | 2.08E-1 | 2.58 | 2.04E-1 | 2.14 | 4.35E-1 |
|  | BioVU | 2.17E-1 | 9.42E-1 | 3.15 | 7.46E-2 | -2.93 | 2.04E-1 |
|  | HRS | -2.21 | 7.50E-1 | 6.00E-2 | 9.90E-1 | -2.26 | 6.00E-1 |
| rs145873257  (16:7759119) | CFS | -4.93 | 2.29E-1 | -5.48 | 5.55E-2 | 9.76E-1 | 7.71E-1 |
|  | ARIC | -1.61 | 5.57E-1 | -1.75 | 2.95E-1 | 5.01E-2 | 9.78E-1 |
|  | WHI | -5.59E-1 | 8.04E-1 | 1.49 | 2.22E-1 | -2.05 | 2.14E-1 |
|  | BioVU | 3.02 | 1.55E-1 | 1.93 | 1.23E-1 | 1.09 | 5.03E-1 |
|  | HRS | -8.08 | 1.00E-2 | -4.75 | 1.00E-1 | -3.33 | 1.00E-1 |
