# Supplementary material for: Rare variants in fox-1 homolog A (RBFOX1) are associated with lower blood pressure
Source: PLoS Genet. 2017 Mar 27;13(3):e1006678. doi: 10.1371/journal.pgen.1006678 (PMC5386302; doi:10.1371/journal.pgen.1006678)
Supplement: S3 Table — a Include both founders and nonfounders (DOCX) [file pgen.1006678.s006.docx]

**S3 Table. Single SNP association analysis for exome array variants of *RBFOX1* identified in CFS.**

| **SNP** | **MAF ^a^** | **MAF ^b^** | **Estimate** | **SE** | **P-Value** | **Function** | **Amino Acid** | **PolyPhen** |
| --- | --- | --- | --- | --- | --- | --- | --- | --- |
| rs149974858 | 3.59E-3 | 0 | -2.22E+1 | 6.92 | 1.55E-3 | Missense | p.Pro38Ala | Probably damaging |
| rs148751394 | 7.17E-4 | 0 | -1.42E+1 | 1.23E+1 | 2.48E-1 | Missense | p.Pro44Thr | Possibly damaging |
| rs151214012 | 7.17E-4 | 2.75E-3 | -1.98E+1 | 1.27E+1 | 1.20E-1 | Missense | p.Pro68Ser | Probably damaging |
| rs145873257 | 8.61E-3 | 1.10E-2 | -4.93 | 4.09 | 2.29E-1 | Missense | p.Gly374Ser | Benign |

^a^ Include both founders and nonfounders

^b^ Founders only; MAF=0 indicates that founders for carriers are not genotyped
