# Supplementary material for: Rare variants in fox-1 homolog A (RBFOX1) are associated with lower blood pressure
Source: PLoS Genet. 2017 Mar 27;13(3):e1006678. doi: 10.1371/journal.pgen.1006678 (PMC5386302; doi:10.1371/journal.pgen.1006678)
Supplement: S4 Table — a Include both founders and nonfounders. b Calculated by the weighted average of Cleveland Family Study, Atherosclerosis Risk in Communities, Women’s Health Initiative, Vanderbilt University Biobank, and Health and Retirement Study. c Calculated by the weighted average of Atherosclerosis Risk in Communities, Family Blood Pressure Program, Africa America Diabetes Mellitus Study, and Howard University Family Study (H.W., unpublished data). d Calculated based on exome array of Nigeria data (H.W., unpublished data). (DOCX) [file pgen.1006678.s007.docx]

**S4 Table. Minor allele frequencies of *RBFOX1* exonic functional variants in white, African-American, and African populations.**

|  | **Whites ^a^** | **African Americans ^b^** | **Africans ^c^** |
| --- | --- | --- | --- |
| rs149974858_G | 5.57E-4 | 0 | 0 |
| rs148751394_T | 1.25E-4 | 0 | 0 |
| rs151214012_T | 9.09E-4 | 3.48E-5 | 0 |
| rs145873257_A | 2.13E-3 | 3.03E-4 | 1.12E-3 |

^a^ Calculated by the weighted average of Cleveland Family Study, Atherosclerosis Risk in Communities, Women’s Health Initiative, Vanderbilt University Biobank, and Health and Retirement Study.

^b^ Calculated by the weighted average of 3300AAs from Atherosclerosis Risk in Communities, 4394 AAs from Family Blood Pressure Program, 2019 AAs from Africa America Diabetes Mellitus Study, and 1727 AAs from Howard University Family Study.

^c^ Calculated based on 2689 individuals from Nigeria.
